# A Multisite Benchmarking Trial of Capnometry Guided Respiratory Intervention for Panic Disorder in Naturalistic Treatment Settings

**DOI:** 10.1007/s10484-017-9354-4

**Published:** 2017-02-13

**Authors:** David F. Tolin, Patrick B. McGrath, Lisa R. Hale, Daniel N. Weiner, Ralitza Gueorguieva

**Affiliations:** 1 Anxiety Disorders Center, The Institute of Living, 200 Retreat Avenue, Hartford, CT 06106 USA; 2 Department of Psychiatry, Yale University School of Medicine, 300 George St., New Haven, CT 06511 USA; 3 AMITA Health - Alexian Brothers Health System, 600 Alexian Way, Elk Grove Village, IL 60007 USA; 4 Kansas City Center for Anxiety Treatment, University of Missouri-Kansas City, 10555 Marty St., Overland Park, KS 66212 USA; 5 Department of Psychology, University of California at Berkeley, Tolman Hall, Berkeley, CA 94720 USA; 6 Department of Biostatistics, Yale University School of Public Health, 60 College St., New Haven, CT 06520 USA

**Keywords:** Panic disorder, Breathing, Biofeedback, Respiration, Hyperventilation, Freespira

## Abstract

Panic disorder (PD) is associated with hyperventilation. The efficacy of a brief respiratory feedback program for PD has been established. The aim of the present study was to expand these results by testing a similar program with more clinically representative patients and settings. Sixty-nine adults with PD received 4 weeks of Capnometry Guided Respiratory Intervention (CGRI) using Freespira, which provides feedback of end-tidal CO_2_ (P_ET_CO_2_) and respiration rate (RR), in four non-academic clinical settings. This intervention is delivered via home use following initial training by a clinician and provides remote monitoring of client adherence and progress by the clinician. Outcomes were assessed post-treatment and at 2- and 12-month follow-up. CGRI was associated with an intent-to-treat response rate of 83% and a remission rate of 54%, and large decreases in panic severity. Similar decreases were found in functional impairment and in global illness severity. Gains were largely sustained at follow-up. P_ET_CO_2_ moved from the slightly hypocapnic range to the normocapnic range. Benchmarking analyses against a previously-published controlled trial showed very similar outcomes, despite substantial differences in sample composition and treatment settings. The present study confirms prior clinical results and lends further support to the viability of CGRI in the treatment of PD.

## Introduction

Hyperventilation and other respiratory abnormalities play a significant role in the etiology or maintenance of panic disorder (PD) (Klein 1993; Ley 1985). Patients with PD show lower end-tidal (exhaled) CO_2_ (P_ET_CO_2_), a marker of hyperventilation, compared to anxious or healthy controls (Meuret et al. 2008; Wilhelm et al. 2001). The acute effects of hyperventilation and compensatory mechanisms include many physiological sensations that are consistent with those seen in anxiety and panic, including gastrointestinal distress, cold sensations, fatigue, rapid or irregular heartbeat, chest pain, impaired breathing, muscle tension, and paresthesias.

Meuret et al. (2008) reported outcomes from capnometry-assisted respiratory training (CART), which measured and provided feedback on P_ET_CO_2_ over 4 weeks. Sustained increases in P_ET_CO_2_ levels and significant reduction in panic symptom severity and frequency were documented.(Meuret et al. 2009) More recently, Meuret et al. (2010) found that panic symptom severity improved significantly and equally with CART and cognitive therapy.

The aim of the present study was to replicate and extend the findings of Meuret et al. (2008) using a novel system, and to benchmark the effectiveness of Capnometry Guided Respiratory Intervention (CGRI) in a clinically representative sample of PD patients seeking treatment in naturalistic clinical settings vs. the academic centers where prior CART studies were performed.

## Methods

### Participants

**Fig. 1:**
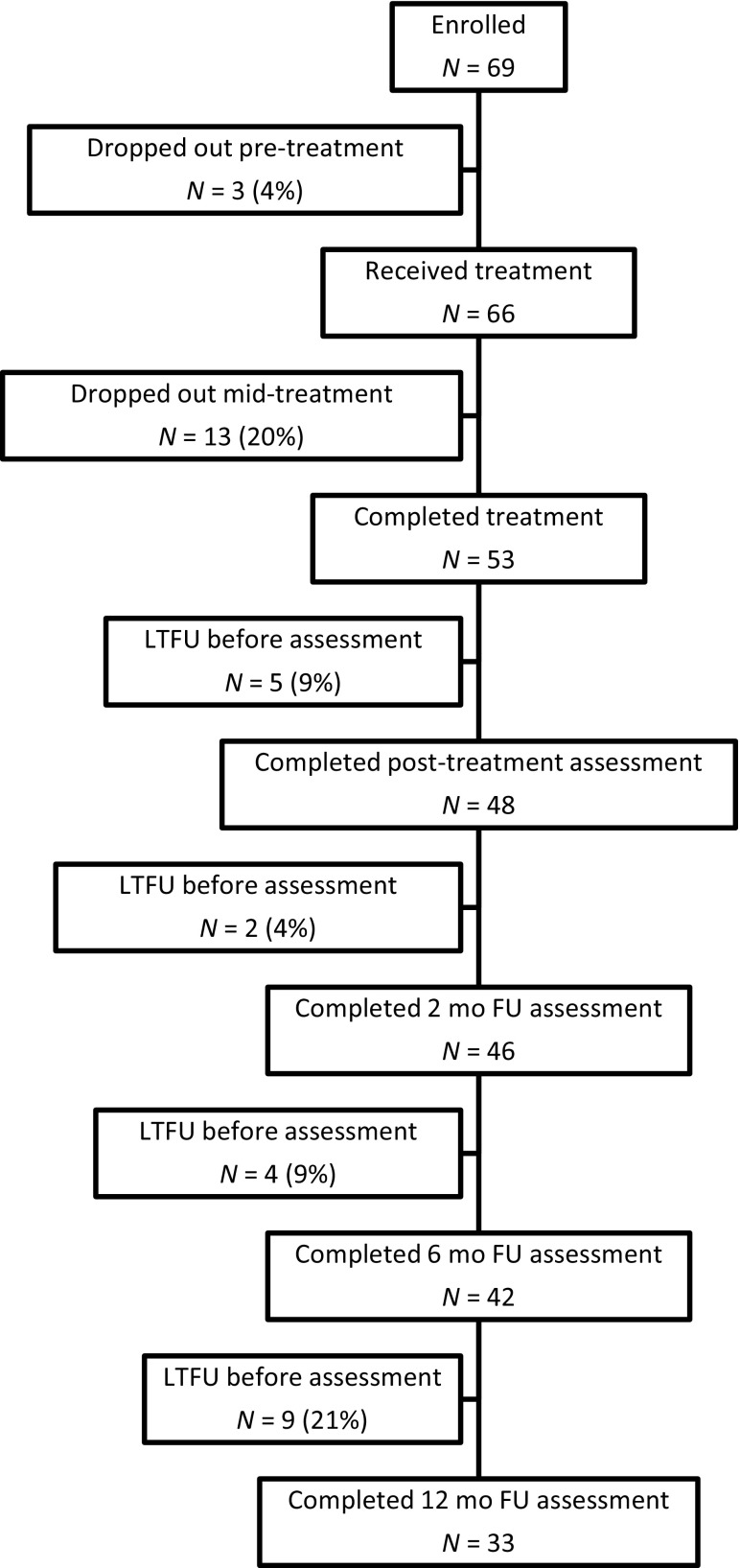
Diagram of participant flow. LTFU—lost to follow-up

Participants were recruited from four geographically diverse non-academic outpatient clinics. Eligible participants had a primary diagnosis of PD, were 18–65 years old, were rated as “moderately ill” or greater on the Clinician Global Impression Scale, and were either off medications or had been stable on medications for at least 3 months. Participants were ineligible if they were pregnant; currently or recently enrolled in another device or drug study; currently receiving other psychological treatment; had been unresponsive to cognitive-behavioral therapy or BR within the past 3 months; or had evidence of organic mental disorder, severe suicidality, psychotic disorder, substance dependence, uncontrolled cardiovascular or pulmonary disease, or seizures. A diagram of participant flow is shown in Fig. 1.

### Measures

Diagnoses were determined using the *Mini International Diagnostic Interview* (MINI) (Sheehan et al. 1998). The primary outcome measure was the clinician-rated *Panic Disorder Severity Scale* (PDSS) (Shear et al. 1997). Secondary outcome measures were the *Clinician Global Impression-Severity Scale* (CGI-S) (Guy 1976), using specific anchor points developed for patients with panic disorder (Pollack et al. 2003), and the *Sheehan Disability Scale* (Leon et al. 1992). *Patient Satisfaction* was assessed with the question “How likely would you be to recommend this treatment to a friend or family member?”; responses were scored from 0 (“would not recommend”) to 4 (“would definitely recommend”). *Panic Attack frequency* over the past week was collected via case report form at each visit. P_ET_CO_2_ and RR levels were examined by calculating (1) the average baseline-stage P_ET_CO_2_ from the first “at home” treatment session; (2) the average baseline-stage P_ET_CO_2_ from the last treatment session; and (3) the average baseline-stage P_ET_CO_2_ from the 2- and 12-month follow-up visits. At each visit, clinicians completed a record of *Adverse Reactions*/*Adverse Events* (AR/AE)s, scored from 0 (No significant functional impairment) to 5 (Significant functional impairment). For benchmarking purposes, we also included the following outcome measures that were used in the Meuret et al. (2008) RCT: the *Anxiety Sensitivity Index* (ASI) (Reiss et al. 1986), the *Mobility Inventory for Agoraphobia* (MI-AAL) (Chambless et al. 1985), and the *Beck Depression Inventory* (BDI) (Beck et al. 1961).

### Apparatus

Capnometry Guided Respiratory Intervention (CGRI) was conducted using Freespira (Palo Alto Health Sciences, Inc., Danville, CA) which consists of a CO_2_ sensor, a Nexus 7 tablet with the Freespira Mobile App, and a nasal cannula. The CO_2_ sensor transmits the P_ET_CO_2_ and respiration rate (RR) values to the tablet, where the app displays the values and instructs the patient visually and audibly how to proceed during the breathing exercises. Data from each treatment session is immediately streamed via the Nexus tablet to a secure server, which allows compilation of aggregate data as well as client-by-client and session-by-session review of adherence and progress. We previously found excellent test–retest reliability in a sample of 11 healthy nonsmokers for RR (*r* = 0.90) and P_ET_CO_2_ (*r* = 0.93).

### Procedure

After providing informed consent, participants completed the pre-treatment measures and met with a study clinician for assessment. CGRI was conducted over 4 weeks. Patients were instructed to perform breathing sessions twice each day at home. Breathing sessions were 17 min long and consisted of a *baseline stage* during which the patient sits quietly and relaxed with eyes closed (2 min), a *pacing stage* during which the patient monitors P_ET_CO_2_ level and RR while breathing with tones at a specified rate (10 min), and a *transition stage* during which the patient maintains breathing pattern without the tones but with continued P_ET_CO_2_ and RR feedback (5 min). For the pacing stage, the tones were set by the therapist for 13, 11, 9 or 6 breaths per minute, representing each progressive week of the program. The mobile app showed patients their current P_ET_CO_2_ level, target P_ET_CO_2_ level (37–40 mm Hg), current RR, and target RR (varying by week).

Participants had four weekly visits with a study therapist to review progress, ask questions, and address any concerns. No other therapeutic activities were conducted. Participants completed the study measures again at mid-treatment and post-treatment. At the end of treatment, participants returned the device and were given no further instructions or contact with research staff. They then returned for follow-up assessment and extended baseline P_ET_CO_2_ monitoring sessions at 2- and 12-month follow-up, as well as a telephone questionnaire regarding panic attacks at 6-month follow-up.

### Data Analytic Plan

For patients with at least one post-treatment data point, PDSS, SDS and CGI-S scores at post-treatment and at 2 and 12-month follow-up were estimated based on previous values using a Markov Chain Monte Carlo approach. The imputation was repeated five times. The corresponding statistical method was applied to each of the five imputed data sets and results were averaged across imputed data sets appropriately accounting for the between and within imputed data set variances.

Response was defined as a 40% or greater reduction in scores on the PDSS; remission was defined as a score of five or less on the PDSS (Furukawa et al. 2009). Proportions of participants with the desired outcome and associated 95% lower bounds were estimated. The proportions were also compared to 50% using a one-sided Wald test. For the continuous outcomes (change from pre-treatment on PDSS, SDS and CGI-S) the mean score was estimated, 95% lower bound was calculated and Cohen’s *d’* within-group effect sizes were calculated.

Moderator analyses were conducted, with change in clinical measures from pre-treatment used as the dependent variable and the potential moderator (hypocapnia defined as baseline-stage P_ET_CO_2_ < 37 vs. normocapnic defined as P_ET_CO_2_ ≥ 37) as a predictor. Means, SDs, 95% CIs, and Cohen’s *d’* effect sizes within each group were calculated. Cohen’s *d* effect sizes for change from pre-treatment in the two groups were also calculated.

Benchmarking analyses were conducted by comparing the present data to those of the CART group in the Meuret et al. (2008) study. Pre-treatment variables were compared between the two studies using Cohen’s *d* for continuous variable and odds ratios (OR) for categorical variables. Changes from pre- to post-treatment in each study were compared using Cohen’s *d*.

## Results

### Sample Description

As shown in Table 1, participants had a mean age of 37 years. Just over half the sample was female, and one quarter was nonwhite. At pre-treatment, PD severity was in the moderate range. The average participant was rated as moderately ill on the CGI, and reported moderate overall functional impairment on the SDS. Mean pre-treatment P_ET_CO_2_ levels were mildly hypocapnic. Compared to Meuret et al.’s (2008) sample, the present participants were more likely to be male, five times more likely to be African-American or Hispanic, over three times more likely to be taking antidepressant or benzodiazepine medications, and more likely to meet criteria for comorbid major depressive disorder. Although PD severity was identical, the present sample reported markedly higher levels of disability, as well as somewhat higher levels of agoraphobic avoidance. The present sample was also less hypocapnic.

**Table 1:** Sample description for the present study and the Meuret et al. (2008) study

	Present sample	Meuret et al. (2008)	*d*	OR
*N*	69	37	–	–
Age *M* (*SD*)	36.6 (11.0)	41.0 (8.9)	−0.44	–
Female *N* (%)	41 (59.4%)	24 (64.9%)	–	0.79
African-American or Hispanic *N* (%)	17 (24.6%)	2 (5.4%)	–	5.72
Duration of Panic Disorder, Years *M* (*SD*)	13.5 (12.2)	8.7 (9.1)	0.44	–
Number of Panic Attacks/Week *M* (*SD*)	2.7 (3.3)	–	–	–
SSRI/SNRI *N* (%)	20 (29.0%)	4 (10.8%)	–	3.37
Benzodiazepine *N* (%)	27 (39.1%)	6 (16.2%)	–	3.32
Comorbid Diagnoses				
Major Depressive Disorder *N* (%)	14 (20.3%)	5 (13.5%)	–	1.63
Posttraumatic Stress Disorder *N* (%)	7 (10.1%)	0 (0%)	–	–
Bipolar Disorder *N* (%)	1 (1.4%)	0 (0%)	–	–
Outcome Measures				
PDSS *M* (*SD*)	2.1 (0.5)	2.1 (0.6)	0.00	–
SDS *M* (*SD*)	5.0 (2.2)	2.5 (2.4)	1.09	–
CGI-S *M* (*SD*)	4.5 (0.7)	–	–	–
P_ET_CO_2_ *M* (*SD*)	36.3 (3.5)	32.16 (4.8)	0.99	–
RR *M* (*SD*)	14.6 (4.4)	11.57 (5.0)	0.64	–
Other Measures				
ASI *M* (*SD*)	2.2 (0.7)	1.9 (0.8)	0.40	–
BDI *M* (*SD*)	12.5 (7.9)	11.2 (8.4)	0.16	–
MI-AAL *M* (SD)	2.40 (0.7)	1.9 (0.6)	0.77	–

*SSRI* Selective serotonin reuptake inhibitor; *SNRI* Serotonin/norepinephrine reuptake inhibitor; *PDSS* Panic Disorder Severity Scale; *SDS* Sheehan Disability Scale; *CGI-S* Clinician’s Global Impression-Severity; P_ET_CO_2−_End-tidal CO_2_; *RR* Respiration rate; *ASI* Anxiety Sensitivity Index; *BDI* Beck Depression Inventory; *MI-AAL* Mobility Inventory for Agoraphobia

### Attrition

Among those who started CGRI, 20% dropped out during the course of treatment. The most common stated reason (*n* = 7) was inability or unwillingness to meet the time commitment of the study. One participant cited lack of perceived efficacy, and another cited adverse effects. The remainder were lost to contact with no explanation given. Another 20 were lost after treatment but before one of the post-treatment or follow-up assessments, with no explanation given. Participants who did and did not complete the treatment did not differ significantly in terms of age, gender, duration of illness, panic severity, agoraphobic avoidance, anxiety sensitivity, depression, functional impairment, global illness severity, or prevalence of comorbid major depressive disorder (all *p*s > 0.05).

### Adherence

Treatment adherence was calculated by determining the proportion of CGRI sessions completed over the course of the study (target = 56), as evidenced by automatic uploads to a cloud-based server. Because some patients had completed more than the required number of respiratory sessions, we coded all patients who completed 56 or more sessions as 100% compliant; for all others, we calculated adherence as the number of completed sessions divided by 56. The average adherence using this calculation was 84.1% (SD = 18%).

### Outcome of CGRI on Panic Disorder Severity

The proportion of responders at post-treatment was 85.4% (SE = 5.1%) in treatment completers, and 83.2% (SE = 5.3%) in the intent to treat (ITT) sample. The rate of remission was 56.3% (SE = 7.2%) in treatment completers, and 54.4% (SE = 6.8%) in the ITT sample. As shown in Table 2, average PDSS decrease from pre-treatment in completers was significant, with a large effect size. Panic severity decreased from the “markedly ill” range to the “slightly ill” range. Thirty-four (70.8%) of the 48 treatment completers reported experiencing no panic attacks in the past week.

**Table 2:** Outcomes on primary and secondary measures

	Pre	Post			2-Month follow-up			6-Month follow-up			12-Month follow-up		
	M (SD)	M (SD)	Est. mean (SE) change	*d’*	M (SD)	Est. mean (SE) change	*d’*	M (SD)	Est. mean (SE) change	*d’*	M (SD)	Est. mean (SE) change	*d’*
Treatment completers													
PDSS	14.8 (3.6)	5.4 (4.4)	9.4 (0.6)	2.2	6.0 (5.2)	8.8 (0.7)	1.8	–	–	–	5.0 (6.2)	9.4 (1.0)	1.7
CGI-S	4.5 (0.7)	2.7 (1.0)	1.8 (0.2)	1.6	2.4 (1.2)	2.1 (0.2)	1.5	–	–	–	2.1 (1.1)	2.3 (0.2)	1.8
SDS	14.4 (6.7)	7.4 (6.7)	7.0 (0.9)	1.2	6.1 (5.6)	8.6 (1.0)	1.3	–	–	–	6.1 (6.6)	8.3 (1.3)	1.1
PA	2.4 (2.6)	0.5 (1.0)	2.0 (0.3)	0.8	0.6 (1.3)	1.8 (0.4)	0.7	0.4 (1.0)	2.2 (0.4)	0.9	0.8 (2.5)	1.6 (0.4)	0.7
P_ET_CO_2_	34.0 (4.6)	38.7 (3.4)	4.8 (0.7)	1.0	37.7 (3.9)	3.7 (0.7)	0.8	–	–	–	37.3 (4.0)	3.5 (0.9)	0.7
RR	14.1 (5.0)	11.4 (5.0)	2.8 (0.9)	0.5	8.6 (2.8)	5.4 (0.7)	1.1	–	–	–	9.3 (3.9)	3.8 (0.9)	0.8
Intent to treat													
PDSS	14.9 (3.6)	5.4 (4.3)	9.5 (0.6)	2.1	6.1 (5.2)	8.9 (0.7)	1.8	–	–	–	5.4 (6.6)	9.5 (0.9)	1.5
CGI-S	4.5 (0.7)	2.7 (1.0)	1.8 (0.2)	1.7	2.5 (1.2)	2.0 (0.2)	1.4	–	–	–	2.1 (1.3)	2.4 (0.2)	1.5
SDS	14.8 (6.6)	7.7 (6.7)	7.1 (0.9)	1.1	6.4 (5.7)	8.4 (0.8)	1.3	–	–	–	5.7 (7.1)	9.1 (1.4)	1.2
PA	2.7 (3.3)	0.5 (1.1)	2.2 (0.4)	0.8	0.7 (1.4)	2.0 (0.4)	0.7	0.3 (1.0)	2.4 (0.4)	0.8	0.9 (2.9)	1.8 (0.3)	0.8
P_ET_CO_2_	34.6 (4.5)	39.0 (3.5)	4.5 (0.6)	1.0	37.5 (3.9)	3.0 (0.8)	0.6	–	–	–	37.3 (4.2)	2.7 (1.1)	0.5
RR	14.4 (5.1)	11.6 (5.2)	2.8 (0.8)	0.5	8.6 (2.8)	5.9 (0.7)	1.2	–	–	–	9.8 (5.0)	4.7 (1.1)	0.6

*PDSS* Panic Disorder Severity Scale; *CGI-S* Clinician’s Global Impression-Severity; *SDS* Sheehan Disability Scale; *PA* Panic attacks in past week; P_ET_CO_2_—End-tidal CO_2_; *RR* Respiration rate

At 2-month follow-up, the proportion of responders was 71.1% (SE = 6.8%) in treatment completers, and 71.8% (SE = 5.6%) in the ITT sample. The rate of remission was 53.3% (SE = 7.4%) in treatment completers and 52.9% (SE = 6.4%) in the ITT sample. Average PDSS decrease from pre-treatment was significant, with a large effect size in completers and in the ITT sample. Thirty-three (71.7%) of the 46 follow-up completers reported experiencing no panic attacks in the past week.

At 12-month follow-up, the proportion of responders was 81.8% (SE = 6.7%) in treatment completers, and 76.5% (SE = 5.7%) in the ITT sample. The rate of remission was 69.7% (SE = 8%) in treatment completers, and 59.4% (SE = 7.3%) in the ITT sample. Average PDSS decrease from pre-treatment was significant, with a large effect size in treatment completers and in the ITT sample. Twenty-six (78.8%) of the 33 follow-up completers reported experiencing no panic attacks in the past week.

### Outcome of CGRI on Secondary Outcome Measures

As shown in Table 2, CGI-S, SDS, and number of panic attacks showed significant and substantial decreases at post-treatment, with large effect sizes in treatment completers and in the ITT sample. CGI-S scores decreased from the moderate range to the borderline to mild range. SDS scores decreased from the moderate range to the mild range. At two-month follow-up, CGI-S and SDS continued to show significant decreases from pre-treatment, with large effect sizes for treatment completers and in the ITT sample. Interestingly, SDS scores decreased even further over the follow-up period. Number of panic attacks per week moderately decreased from pre-treatment in treatment completers and in the ITT sample. At 6-month follow-up, the number of panic attacks was substantially decreased from pre-treatment for treatment completers and the ITT sample. At 12-month follow-up, CGI-S and SDS continued to show significant decreases from pre-treatment, with large effect sizes for treatment completers and the ITT sample. SDS scores were in the mild range of impairment. Number of panic attacks per week were moderately decreased from pre-treatment.

### Outcome of CGRI on Respiratory Parameters

As shown in Table 2, average increase in baseline-stage P_ET_CO_2_ from first “at-home” treatment to last treatment was significant, with a large effect size in both treatment completers and the ITT sample. P_ET_CO_2_ levels increased from the mildly hypocapnic range to the normocapnic range. Average decrease in baseline-stage RR from first “at-home” treatment to last treatment was significant, with a moderate effect size in treatment completers and the ITT sample. RR decreased from the middle of the normal range to the lower end of the normal range.

At two-month follow-up, average increase in in baseline-stage P_ET_CO_2_ from first “at-home” treatment was significant, with a moderate effect size in treatment completers and the ITT sample. The corresponding average decrease in baseline-stage RR was significant, with a large effect size in treatment completers and the ITT sample.

At 12-month follow-up, average increase in P_ET_CO_2_ from first “at-home” treatment was significant with a medium effect size in treatment completers and the ITT sample. The corresponding average baseline-stage decrease in RR was significant with a large effect size in treatment completers and a medium effect size in the ITT sample.

### Moderator Analysis

The 62 patients who completed at least one breathing assignment were split between 39 hypocapnic and 23 normocapnic participants based on P_ET_CO_2_ values from their first “at-home” session. In a completer analysis of PDSS reduction during treatment and follow-up, the hypocapnic and normocapnic groups did not differ significantly at post-treatment (*t*_46_ = 1.2, *p* = 0.2, *d* = 0.4) but differed significantly at 2-month follow-up (*t*_43_ = 2.5, *p* = 0.02, *d* = 0.8); Mean PDSS decrease for hypocapnic patients was 9.9 (SD = 8.1); for normocapnic patients it was 6.2 (SD = 3.5). At 12-month follow-up the difference was again not significant (*t*_43_ = 0.5, *p* = 0.6, *d* = 0.2). A moderating effect of pre-treatment hypocapnia on change in P_ET_CO_2_ from pre-treatment was found; this difference was significant at post-treatment and at 2-month follow-up, and marginal at 12-month follow-up (post-treatment: *t*_45_ = 2.8, *p* = 0.007, *d* = 0.9; 2-month follow-up: *t*_42_ = 2.6, *p* = 0.01, *d* = 0.9; 12-month follow-up: *t*_26_ = 2.1, *p* = 0.05, *d* = 0.9) The increase in P_ET_CO_2_ was greater for hypocapnic participants than for normocapnic participants. Results for the ITT sample were very similar.

### Patient Satisfaction

At post-treatment, the mean response to the question “How likely would you be to recommend this treatment to a friend or family member?” (0–4) was 3.50 (SD = 0.77), with 88% responding positively (score 3 or 4). At 2-month follow-up, the mean response was 3.53 (SD = 0.73), with 87% responding positively; at 12-month follow-up the mean response was 3.33 (SD = 0.82) with 82% responding positively.

### Adverse Events

There were no Serious Adverse Events (SAEs). The overall rate of moderate-to-severe dizziness (score 3–5) for all visits was 2% (6 reports from 319 patient visits). For moderate-to-severe lightheadedness the rate was also 2% (7/319). The highest rates of moderate-to-severe dizziness or lightheadedness were seen in the first treatment visit: 4% for dizziness (2 reports from 56 first visits) and 7% for lightheadedness 4/56). One patient reported significant functional impairment due to dizziness (score = 5) after visit 2. Another patient reported significant functional impairment (score = 5) due to lightheadedness after visit 1. There were no reports of moderate-to-severe dizziness or lightheadedness after visit 4. Reported “Other” AR/AEs included nausea (3), fatigue (1), tingling in hands, mouth or ears (2), shakiness (1) and dry mouth (1). Three panic attacks during sessions were reported. None of these “Other” AR/AEs resulted in significant impairment and all ceased once the session was completed.

### Benchmarking Outcomes

Table 3 shows results of the present sample vs. those in Meuret et al.’s (2008) CART study. Because there was no attrition in the Meuret study, the treatment completer and ITT outcomes are the same. We used our completer analyses in order to obtain the most comparable statistics. We obtained a PDSS reduction nearly identical to that of Meuret et al. (2008) Reductions in SDS and ASI were also very similar, although it is noted that the present sample exhibited somewhat smaller reductions in MI-AAL and BDI than did the Meuret et al. (2008) sample. Interestingly, P_ET_CO_2_ increase was greater in the present sample despite having higher mean P_ET_CO_2_ levels at pre-treatment.

**Table 3:** Benchmarking the present outcomes against those of the Meuret et al. (2008) study

	Meuret et al. (2008)			Present study*		
	Pre M (SD)	Post M (SD)	*d*_pre−post_	Pre M (SD)	Post M (SD)	*d*_pre−post_
PDSS	2.1 (0.6)	0.7 (0.4)	2.6	2.1 (0.5)	0.8 (0.6)	2.3
SDS	2.5 (2.4)	0.7 (0.9)	0.9	4.8 (2.2)	2.6 (2.3)	1.0
ASI	1.9 (0.8)	0.9 (0.6)	1.3	2.2 (0.6)	1.5 (0.8)	1.0
MI-AAL	1.9 (0.6)	1.4 (0.5)	0.8	2.3 (0.7)	2.0 (0.7)	0.4
BDI	11.2 (8.4)	4.2 (3.5)	1.1	11.8 (6.9)	7.5 (7.3)	0.6
P_ET_CO_2_	32.2 (4.8)	34.6 (5.0)	0.5	36.3 (0.4)	38.7 (3.0)	1.1
RR	11.6 (5.0)	9.3 (4.1)	0.5	14.2 (3.9)	12.0 (4.4)	0.5

To facilitate comparison with the Meuret et al. study, PDSS and SDS scores in the present sample were converted to mean scores rather than sum scores as used in the primary analyses

*PDSS* Panic Disorder Severity Scale; *CGI-S* Clinician’s Global Impression-Severity; *SDS* Sheehan Disability Scale; *PA* Panic attacks in past week; P_ET_CO_2_—End-tidal CO_2_; *RR* Respiration rate

*Means and standard deviations are shown for the completer sample

## Discussion

The present study confirms prior results supporting the utility of P_ET_CO_2_ feedback in the respiratory treatment of PD. Patients showed a significant decrease in PD severity over 4 weeks of largely home-based treatment. It is encouraging that despite the brevity of the active intervention (4 weeks), gains appear to be largely sustained after treatment discontinuation, with high rates of response and remission obtained over a 12-month period. Importantly, the present study demonstrates the capacity of results from an RCT, conducted in an academic setting, to translate to more typical clinical settings and patients. The present study was substantially more ethnically diverse, with more depressive comorbidity and medication use, than were those in the original CART RCT (Meuret et al. 2008). Unlike the Meuret trial, which was conducted in an academic setting with a single clinician who is an expert in PD and the developer of CART, the present study was conducted at multiple non-academic clinical sites, with several different clinicians at varying levels of expertise. Despite these differences, we obtained a reduction in PD severity that was nearly identical to that obtained in the RCT.

The primary limitation of the present trial is the absence of a control group; as such, these results cannot be considered a definitive documentation of efficacy. The waitlist-controlled trial by Meuret et al. (2008) provided initial evidence of efficacy, while the present study extends those findings to document feasibility and utility in more naturalistic treatment settings.

The extent of treatment moderation awaits further exploration. We found that patients who were hypocapnic at pre-treatment exhibited a greater increase in P_ET_CO_2_ at post-treatment and at follow-up than did patients who were normocapnic at pre-treatment. However, the extent to which this translates into different clinical outcomes is unclear. In the present study, as in the initial trial by Meuret et al. (2008), the intervention appeared equally effective for normocapnic and hypocapnic patients at post-treatment.

The potential practical benefits of CGRI are many. Given the barriers to receiving other forms of empirically-supported therapy for PD, the fact that CGRI is a non-pharmacologic approach that can be made widely available at a relatively low cost makes it particularly desirable. Patient compliance was high, as was patient satisfaction. Adverse events were fairly rare, and generally limited to mild dizziness or lightheadedness in the initial training sessions. The system allows therapists to monitor patients’ progress remotely, using a secure server, thus potentially allowing them to treat a larger number of patients over a wider geographic area. When these factors are considered along with the strong treatment response in the present sample and in previous research, CGRI merits consideration as a treatment option for PD.
